# Easy does it: Selection during interactive search tasks is biased towards objects that can be examined easily

**DOI:** 10.3758/s13414-025-03083-w

**Published:** 2025-05-12

**Authors:** Haden Dewis, Cheryl D. Metcalf, Martin B. Warner, Richard Polfreman, Hayward J. Godwin

**Affiliations:** 1 University of Southampton, Southampton, UK; 2 School of Psychology, University of Southampton, Highfield, Southampton, Hampshire SO17 1BJ UK; 3 Department of Psychology, University of Southampton, Southampton, UK

**Keywords:** Interactive search, Visual search, Guided search, Attentional selection

## Abstract

It is well understood that attentional selection is required to deploy visual attention to relevant objects within displays during visual search tasks. Interactive search, an extension of visual search, refers to tasks wherein an individual must manipulate items within their environment to uncover obscured information whilst searching for a target object. Here, we conducted two independent interactive search experiments where participants were asked to interact with virtual cubes to locate a target *T* shape embedded onto the side of one of the cubes. Our goal here was to investigate the drivers of attentional selection within interactive searches. To do so, we manipulated the effort required to rotate cubes (Experiment 1) and the quantity of shapes attached to the cubes (Experiment 2). Our findings suggest that the perceived effort required to interact with an object is an extremely strong driver of attentional selection within interactive search behaviors. Here, targets may be slower to be detected when that target is obscured within or by an object that conveys, in some shape or form, greater difficulty to examine compared with other objects. These findings provide an exciting first step towards understanding the factors that influence selection during interactive searches. Data and experimental code for all experiments in this study can be accessed online via this web address: https://osf.io/2zyvf/?view_only=ae4f4f2c36ab4e6aae5da3e99fb81988. Experiments were not preregistered.

You are late for work, and you cannot remember where you placed your car keys. You start rummaging through your desk, picking up books, moving piles of notes, frantically trying to find where you left them. This scenario is an example of an interactive search task: A task wherein the observer must manipulate items or physically change their viewing position to uncover hidden or obscured information whilst searching for a target (Sauter et al., 2020). A handful of studies have investigated interactive search in detail, ranging from simple tasks, such as searching for marbles (Gilchrist et al., 2001) or LEGO^®^ bricks (Hout et al., 2022; Sauter et al., 2020), to more societally important and complex tasks, such as police personnel searching through houses for drugs and weapons (Riggs et al., 2017, 2018). Interactive searches are not limited to only the physical domain but are commonplace in virtual environments as well where individuals will typically manipulate and change visual displays (Drew et al., 2013; Godwin et al., 2024; Solman et al., 2012, 2013).

The study of interactive search is an extension of visual search, one of the most extensively studied tasks in cognitive psychology (Chan & Hayward, 2013; Wolfe, 2020). In visual search, it has long been known that it is impossible to process all items within the visual field at once, and instead visual attention must be deployed to subsets of objects in the visual display. Early models of visual attention characterized this process as a dichotomy between top-down and bottom-up control (Corbetta & Shulman, 2002; Itti & Koch, 2000; Wolfe, 1994). Here, top-down input describes the current goals of the searcher (e.g., searching for a red object) and bottom-up input describes the physical salience of a stimulus (e.g., a bright object amongst dull objects, a horizontal line amongst vertical lines, and so forth).

It was widely believed that these top-down and bottom-up inputs combined to create an attentional “priority map” which dictated where visual attention should be deployed (Wolfe, 1994). However, later research made it clear that the deployment of visual attention could not be entirely explained via this top-down/bottom-up dichotomy. Summarizing the issues relating to this, Awh et al. (2012) argued that models at the time were too simplistic to account for scenarios wherein attentional selection biases could not be attributed to either top-down or bottom-up control. As such, they presented a model that included new sources of input to the attentional selection process. These new sources were grouped together under the heading of “selection history”, a new category to account for the effects of priming (e.g., Maljkovic & Nakayama, 1994) and reward (Anderson et al., 2011; Hickey et al., 2010a, b, 2015) on attentional control. More recently, Wolfe (2021) expanded upon these sources of input and suggested that attentional control is influenced by five factors: top-down control, bottom-up control, history (priming), value (reward), and scene guidance. Here, scene guidance refers to the utilization of previously learned semantic knowledge about the world to guide visual attention away from areas where targets are unlikely to be (e.g., Henderson & Hayes, 2017; Le‐Hoa Võ & Wolfe, 2015; Pedziwiatr et al., 2021; Võ et al., 2016; Võ & Wolfe, 2013; Wolfe et al., 2011). Overall, it is now generally understood that in addition to top-down and bottom-up control, many factors work in tandem to influence attentional selection via a priority map (Godwin et al., 2014; Wolfe, 2021; Wolfe & Horowitz, 2017).

There is, to our knowledge, no past research examining the factors that influence selection during interactive searches. Here, our goal was to test whether two new sources of input could guide selection during interactive search. These two new sources of input were *physical effort* and *patch value.* Here, physical effort refers to the energetic expenditure required to interact with objects, and patch value is an established term within the foraging literature that describes the perceived value assigned to different objects/areas containing resources (e.g., Charnov, 1976).

We examined these two new sources of input by conducting two interactive search experiments using a novel methodology wherein participants interactively searched for a target *T* shape amongst distractor *L* shapes embedded onto the sides of a set of virtual cubes. Participants interacted with cubes by rotating them with their computer mouse. Here, when a participant clicked on a cube and simultaneously dragged their cursor across the display, the selected cube rotated in the direction of the cursor movement. To ensure effortful interaction, cubes only rotated during cursor movements. In Experiment 1, we manipulated physical effort by making 50% of the cubes “heavy” to rotate and 50% “light”. In Experiment 2, the resources used to influence patch value was the number of shapes embedded onto the sides of the cubes. Here, 50% of cubes were made to be “information-rich” by embedding a shape onto each cube face and 50% were made to be “information-poor” by embedding a single shape onto only one of their six possible faces. Across both experiments, we utilized color to encourage participants to form associations between different cube types (i.e., assigning a blue color to heavy cubes and a yellow color to light cubes in Experiment 1, or a green color to information-rich cubes and a pink color to information-poor cubes in Experiment 2). Examples of trials from both experiments are depicted in Fig. 1, and demonstrations of the experiments with a small number of trials can be found here https://jatos.psychology.soton.ac.uk/publix/kCXBjTn2jof.

**Fig. 1:**
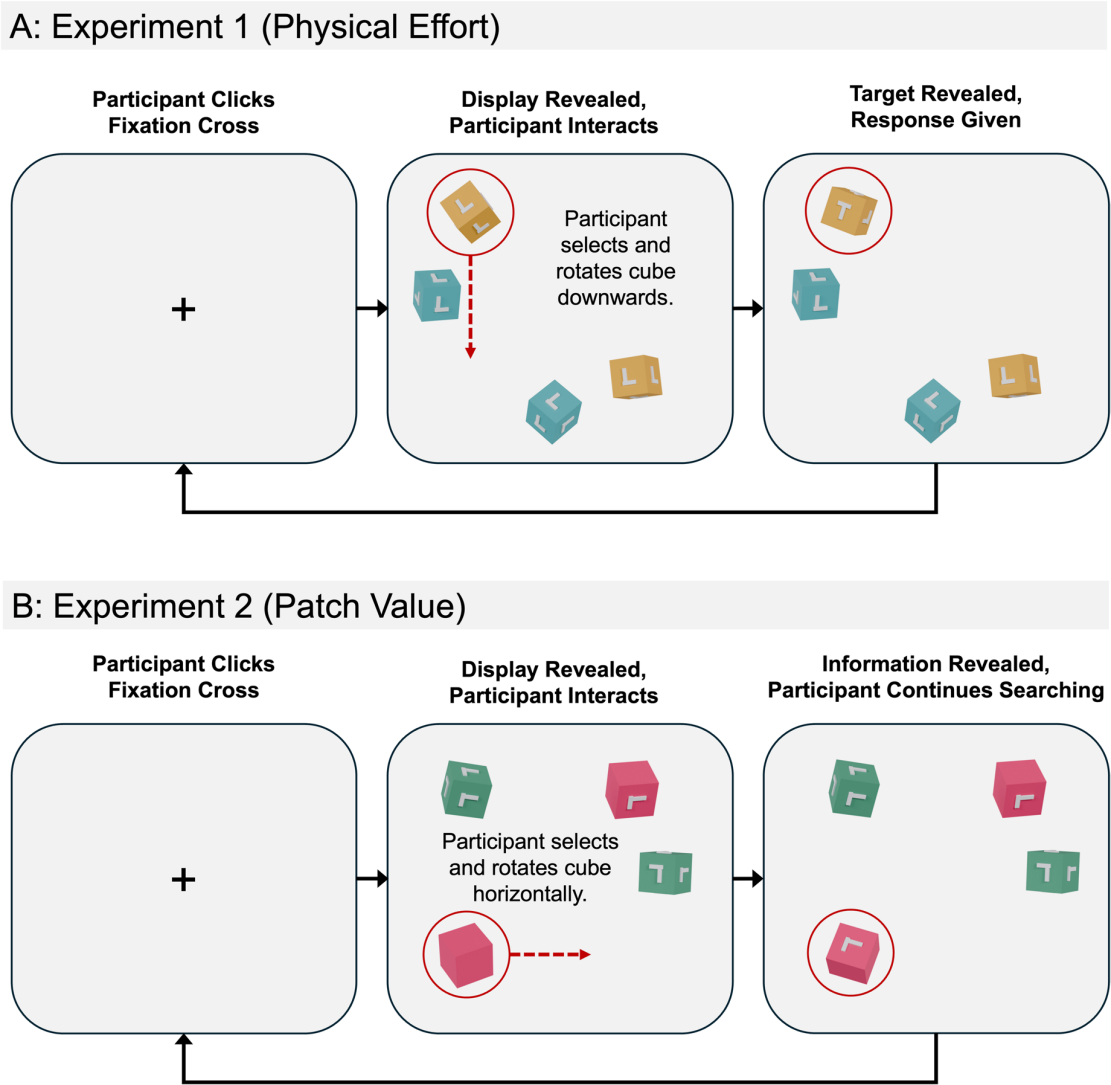
*Trial Structure and Procedure for Experiment *1* and Experiment *2*. Note*. Figure depicts the procedure of a typical trial for both Experiment 1 and Experiment 2. The red circles and arrows were not visible to the participant and are included here to aid visibility. Participants used their cursor to click on a fixation cross presented in the middle of the screen to start a trial. The display was then revealed, and participants then interacted and rotated cubes using their cursor. Once the target was found (or deemed absent), the participant ended the trial with a keyboard press. This whole process then repeated for 120 trials. (Color figure online)

## Physical effort

In visual search tasks, very little physical effort is required to search. Eye movements are the most common of all behaviors (Bargary et al., 2017) and require very little energetic expenditure to conduct (Araujo et al., 2001). In contrast, interactive search tasks often require energetic expenditure via body movements; typically, the upper limbs, as individuals manually manipulate objects with their hands. It is well established that individuals will try to minimize engaging in tasks that require high energetic expenditure (Anderson et al., 2025; Klein-Flügge et al., 2016; Kurniawan et al., 2010; Prévost et al., 2010). Indeed, perceived physical effort has been shown to further influence engagement in safe versus dangerous work practices (Wickens, 2014), the limbs used to reach and grab objects (Morel et al., 2017; Wang et al., 2021), and the decisions made in complex workspaces such as airplane cockpits (Steelman et al., 2011; Wickens, 2015).

In the context of interactive searches, reducing the number of high-effort tasks one engages in is logical, given that there are two dissociable cognitive processes that must work in tandem when doing so: the *action system* for body movements and the *identification system* for target detection (Goodale & Milner, 1992; Jeannerod, 1994; Solman et al., 2012). In cases where high physical effort is involved, more resources must be provided to the action system, thus likely impairing the identification system. This is neatly highlighted in a study by Park et al. (2021). Here, participants were asked to engage in a visual search task whilst simultaneously gripping a “handgrip” device. Whilst searching, participants had to either grip the device with a high grip force (analogous to high physical effort) or a low grip force. In high-grip-force trials, participants were more prone to interreference from distractors in comparison with the low-grip-force trials. Overall, then, it seems likely that in scenarios where the likelihood of uncovering a target is equal between objects, attentional deployment should be biased towards objects that indicate a low level of physical effort to interact with.

## Patch value

Patch value is an established term within the foraging literature, and it describes the perceived value assigned to different areas containing resources (e.g., Charnov, 1976). The foraging literature has historically focused on the criteria animals and individuals use to determine when to leave the current patch that they are obtaining resources from (Charnov, 1976; Ehinger & Wolfe, 2016; Wolfe, 2013; Zhang et al., 2017). The perceived value assigned to specific patches can be influenced by a range of factors (e.g., Bettinger & Grote, 2016; Charnov, 1976; Eliassen et al., 2009; Norberg, 1977), including the quantity of resources one can obtain from a specific patch (Bremset Hansen et al., 2009; Fryxell, 1991; Van Beest et al., 2010). Here, greater value is given to patches containing large quantities of resources (Bremset Hansen et al., 2009).

This bears particular importance when considering interactive search tasks. Here, we propose that interactive search tasks can be further conceptualized as a form of foraging task where searchers must make decisions regarding patch value (Bella-Fernández et al., 2022; Nahari & El Hady, 2025). When searching, individuals must manipulate and move objects to reveal either other obscured items or sections currently not visible to the searcher (i.e., inside an object, behind an object, and so on). However, instead of foraging for food, searchers here are instead foraging for visual information (Nahari & El Hady, 2025). As such, it seems likely that should searchers attempt to be optimal in their search strategy and follow the same rules found within the foraging literature (e.g., Ehinger & Wolfe, 2016), then they should bias their searches towards areas that are resource-rich and capable of providing large quantities of information to the searcher. Likewise, this also makes sense at a probabilistic level: If one does not know where a target may appear, focusing on areas with the largest number of potential targets would be a far more efficient strategy than focusing on those areas with only a small number of potential targets.

## Experiment 1

In Experiment 1, we started by investigating the role of physical effort within interactive search. Based on the previously mentioned literature, we predicted that within our experiment, due to the increased physical effort required to rotate heavy cubes, participants would become more likely to examine the light cubes first within each trial. Likewise, due to the increased effort associated with heavy cubes, we further predicted that participants would become less exhaustive in their searching of heavy cubes, as evidenced by a reduction in the number of cube faces a participant revealed and viewed throughout a trial.

### Method

#### Ethical approval

Ethical approval was given for Experiment 1 by the University of Southampton’s Ethics Committee on 26 September 2023 (ERGO NUMBER: 95398.A1).

#### Participants

A priori power analyses were conducted using the *simr* packing in R (Green & MacLeod, 2016) on pilot data from 15 participants. To avoid the issues associated with “observed power” (see Hoenig & Heisey, 2001, for an explanation), target effect sizes were based on prior research. Power analyses were conducted for each dependent variable being analyzed, and a minimum sample size of 35 participants was recommended to obtain a power level of 0.80.

A total of 40 participants were recruited from the University of Southampton. Of this sample, ages ranged from 18 to 21 years (*M* = 18.88, *SD* = 0.99). Seventy-five percent of the sample were women, 22.50% were men, and 2.50% were nonbinary.

#### Stimuli and apparatus

Stimuli were created using the open-source software Blender (Hess, 2010). Displays consisting of these stimuli were then generated using Three.js (an open-source JavaScript library for displaying three-dimensional graphics within web browsers; Danchilla, 2012) and embedded into a standard jsPsych framework (an open-source JavaScript library for building web-based psychological experiments; De Leeuw, 2015).

The stimuli for Experiment 1 consisted of four different types of virtual cubes: heavy and light cubes containing an *L* distractor shape on each of their six faces, and heavy and light cubes containing a distractor *L* shape on five of their six faces and a single target *T* shape on the remaining sixth face. Heavy and light cubes were assigned a single independent color at the start of the experiment, which did not change throughout the remainder of the experiment. This was to ensure that participants would associate a specific cube color with the effort required to rotate them. Colors were selected from a list of 16 ordered colors used in previous visual search experiments (e.g., Menneer et al., 2007; Stroud et al., 2012). Each consecutive color was approximately equally spaced from the previous in CIE xyY space. The color chosen for heavy cubes was randomized for each participant to reduce any risk of biases towards specific colors. The color selected for the light cubes was always eight steps away from the previously selected heavy cube color in the color list. This ensured that colors were as maximally different as possible to help strengthen the association between color and effort.

**Fig. 2:**
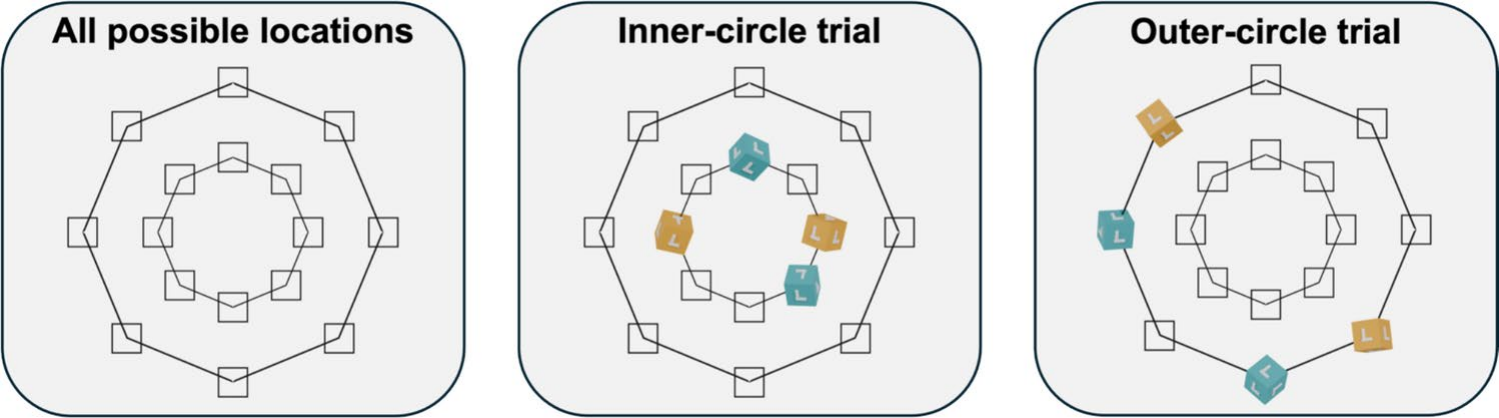
Cube-placing procedure. *Note*. Figure depicts all the possible locations that cubes could have been placed for each trial. The outlines of the circles and locations were not visible to the participant. Participants had to use their cursor to click on a fixation cross presented in the middle of the screen before a display was revealed; this was to ensure that their cursor starting position would be from the center of the screen for each trial. (Color figure online)

Each search display contained two heavy and two light cubes, each of which were randomly assigned to one of eight possible locations on the screen and then randomly rotated through each of their axes by up to 360º. As shown in Fig. 2, on each trial, cubes were placed within one of two concentric circles (inner or outer) each of which contained eight equidistant locations. A single trial could not contain cubes in both the inner and outer circles simultaneously. This was to ensure that the distance from the center of the screen to each cube was equal at the start of the trial to reduce the likelihood that participants would simply interact with the cube closest to the center. Fifty percent of trials were inner-circle trials and 50% were outer-circle trials. The order in which trials were presented was randomized.

Participants completed the study using their own computers or laptops. They were informed to press the *M* key of their keyboard if they believed that the display contained a target and the *Z* key if they believed the display did not contain a target. Participants interacted by clicking on a cube and simultaneously dragging their cursor across the screen. Here, when the participant clicked and dragged their cursor, the selected cube rotated in the direction of the cursor movement. A cube only rotated whilst the participant was clicking and dragging.

#### Design and procedure

Once participants consented to taking part, they were provided with detailed instructions on what was required to complete the experiment, followed by a training segment wherein participants could learn and practice how to rotate cubes with their computer mouse. Following this, participants completed five practice trials with accuracy feedback before starting the real trials, which contained no feedback. All participants then completed a total of 120 trials. Before each trial, participants had to click a fixation cross to reveal the display. The display then remained on screen until the participant made a response to end the trial. Following a participant response, the next trial’s fixation cross was displayed, and the process repeated. This process is depicted in Fig. 1A.

A target cube was present on 50% of the 120 search trials and absent on the remaining trials. The order in which participants completed trials was randomized. Within each trial, two of the four cubes were always substantially more difficult to rotate than the other two cubes. This was achieved by reducing the cursor sensitivity whenever a participant interacted with these heavy cubes, resulting in the cubes rotating at a slower rate and thus requiring ~2–3 times the number of clicks and drags to rotate them by the same magnitude as a light cube. The same color contingencies were used during the practice trials as were during the real trials. All stimuli were evenly split between heavy and light conditions.

### Results

#### Data cleaning

Before any analyses, all data underwent preplanned cleaning procedures based on those used in prior online search experiments (Godwin et al., 2024; Godwin & Hout, 2023). A breakdown for the number of participants/trials removed at each stage of cleaning can be found in Table 1.

**Table 1:** Data cleaning steps for each experiment

	Experiment 1 (effort manipulation)		Experiment 2 (information manipulation)	
Removal step	Trials removed	Remaining trials	Trials removed	Remaining trials
Raw data	0 (0.00%)	4,800 (100%)	0 (0.00%)	5,400 (100%)
Fast/Slow trials	20 (0.42%)	4,780 (99.58%)	1 (0.02%)	5,399 (99.98%)
Guessing trials	11 (0.23%)	4,769 (99.35%)	2 (0.04%)	5,397 (99.94%)

*Note*. Fast trials = trial response times <250 ms; slow trials = trial response times >60,000 ms; “guessing trials” refers to target-present trials in which participants responded present yet never revealed the face of the cube containing the target

No participants were removed from either dataset

First, trials shorter than 250 ms or longer than 60 s were removed from the dataset. The upper limit of this criterion was decided upon from what we deemed to be an acceptable time to have exhaustively checked all four cubes. Likewise, it was implausible that a participant would be able to engage with the array and respond in under 250 ms. Finally, any trials where a participant had responded that the target was present but never revealed the face of the cube containing the target *T* shape were removed from the dataset.

After all cleaning steps, the final dataset consisted of 4,769 trials from 40 participants.

#### Analytic approach

All effects were modelled through Bayesian generalized linear mixed-effects models (BGLMM) via the *brms* package in R (Bürkner, 2017; R Core Team, 2023). The reliability of effects was confirmed using Bayes factors, calculated via the *bayestestR* package in R (Makowski et al., 2019). Bayes factors greater than 1.00 indicate stronger evidence towards the alternative hypothesis, and Bayes factors less than 1.00 suggest stronger evidence towards the null hypothesis. For the purpose of the discussion, we have deemed an effect to be trustworthy if both its 95% credible interval (CI) did not pass through zero, and it possessed a Bayes factor of greater than 3.20.

Where relevant, models used the following fixed factors: presence (absent, present), trial index (a continuous value used as a measure of time through experiment), effort type (heavy, light), and next closest object type (heavy, light). Across all analyses, trial index was rescaled and centered to improve model fitting and interpretation (Kreft et al., 1995). Each model included random intercepts and slopes for participant ID and presence. This allowed for individual variation between participants and trial types within each model.

The likelihood of a participant selecting a light cube was calculated by coding any interaction with light cubes as a 1 and any interactions with heavy cubes as a 0. This was then modelled using a Bernoulli distribution with a logit link function. Likewise, a Poisson distribution was used to model the total number of cube faces viewed by participants. Our analyses focused only on participants’ first and second interactions. Our reason for doing so was that since each trial contained only four cubes, the third and fourth interactions were typically a mirror of the first and second interactions. For example, if a participant’s first two interactions were to the two light cubes, then their remaining interactions would be to the two remaining heavy cubes and vice versa.

Each model was fitted using four chains, with 11,000 iterations and 1,000 warmup iterations to allow for accurate Bayes factors (Makowski et al., 2019). All Gelman–Rubin statistics were below 1.10 for all parameters and visual inspection of the chains indicated good mixing.

#### Response accuracy and response times

Overall, for target-absent trials, participants had high accuracy with few false alarms (*M* = 0.98, *SD* = 0.13) and completed the trials within a reasonable time (*M* = 20,609.03 ms, *SD* = 7,388.47 ms). For target-present trials, participants had good accuracy (*M* = 0.92, *SD* = 0.27) and completed trials within a reasonable time (*M* = 9,775.11 ms, *SD* = 7,201.42 ms). We carried out no further analyses on response accuracy or response times. Our remaining analyses focused on the order of interactions and search exhaustiveness.

#### First interaction choice

**Fig. 3:**
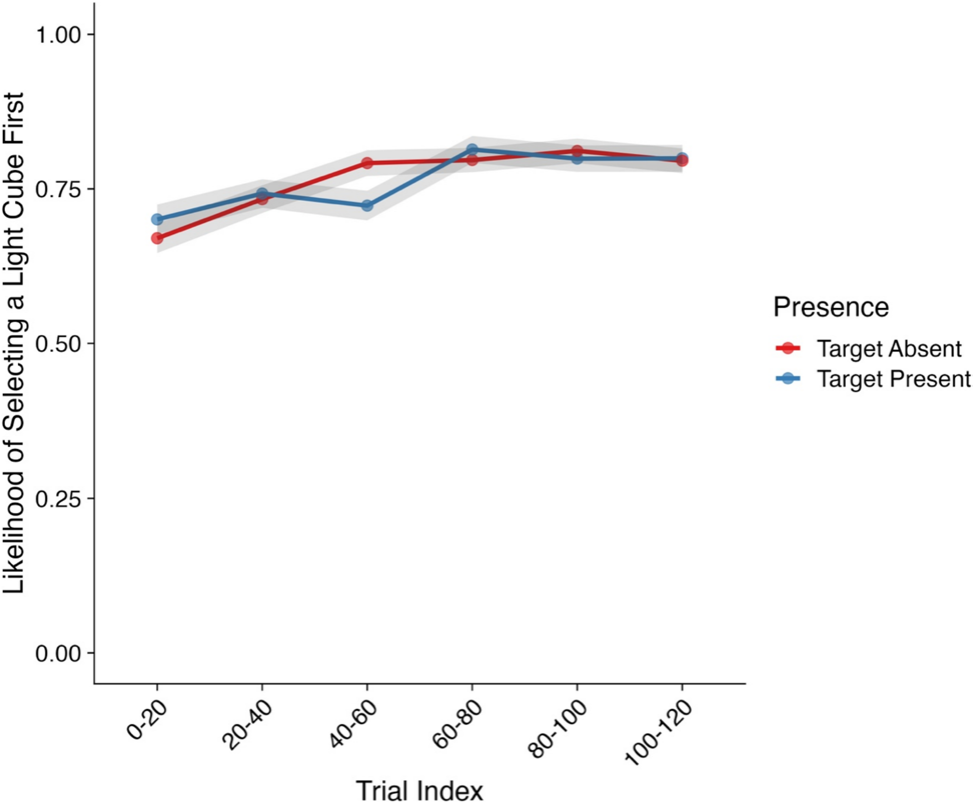
Likelihood of selecting a light cube first (Experiment 1). *Note*. Shaded areas represent ±*SE*. Trial index has been binned into increments of 20 trials for the purposes of visualization only. *SE* = standard error. (Color figure online)

Our first analysis focused on the likelihood of a participant selecting a light cube as their first interaction of a trial. Model effects and their corresponding CIs and Bayes factors can be found in Table 2 and descriptive statistics in Fig. 3.

**Table 2:** Model effects and Bayes factors—Likelihood of selecting a light cube first

*Parameter*	*Estimate*	*CIs*	*R-Hat*	*BF*
Intercept	1.04 (0.22)	**0.62, 1.47**	1.00	**6.58×10**^**3**^
Presence (Absent–Present)	0.02 (0.15)	−0.29, 0.32	1.00	0.16
Trial Index	0.52 (0.08)	**0.37, 0.68**	1.00	**5.16×10**^**5**^
Presence × Trial Index	−0.08 (0.16)	−0.38, 0.23	1.00	0.17

*Note*. CIs = credible intervals; BF = Bayes factor; bolded CI values = CIs that did not pass through zero; bolded BF values = BF >3.20. Values in parentheses represent the associated standard error values. Effects were deemed reliable if CIs did not pass through zero and BF >3.20

We observed a strong main effect of trial index on the likelihood of a participant selecting a light cube first, with no other effects emerging. Here, participants became substantially more likely to select a light cube first as the experiment progressed. As can be seen in Fig. 3, across the first 20 trials, participants’ likelihood of selecting a light cube first was ~0.67 for target-absent trials and ~0.70 for target-present trials. By the time participants reached the end of the experiment, this likelihood increased substantially to ~0.80 for both target-absent and target-present trials.

#### Second interaction choice

**Fig. 4:**
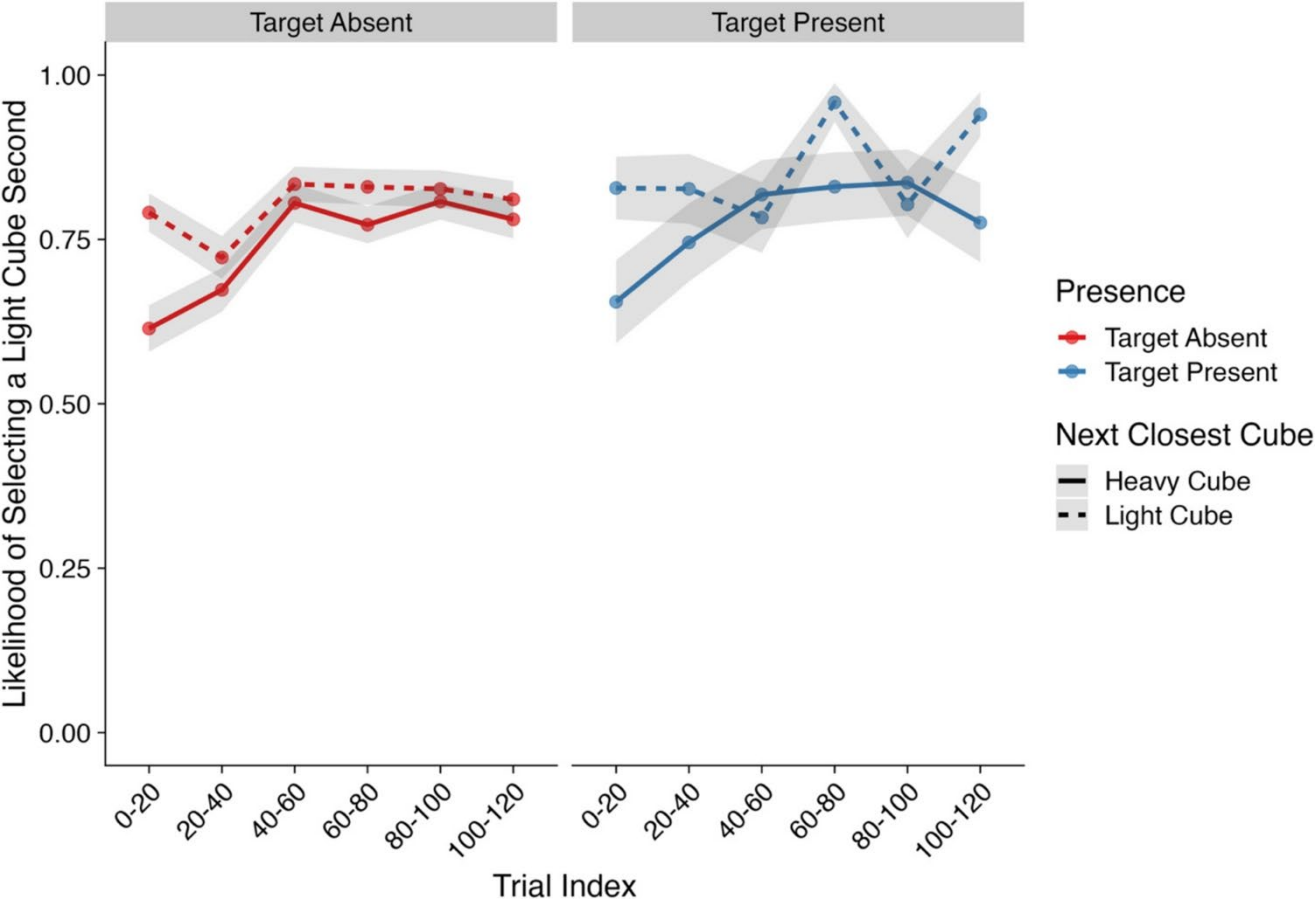
Likelihood of selecting a light cube second (Experiment 1). *Note*. Shaded areas represent ±*SE*. Trial index has been binned into increments of 20 trials for the purposes of visualization only. *SE* = standard error. (Color figure online)

Our next analysis focused on the likelihood that the second cube a participant examined would also be a light cube. Here, we focused only on trials where participants did not find the target within their first interaction. At the start of each trial, the participant had to click on a central fixation cross to reveal the display. As such, a participant’s attention should have been focused on the center of the display (Anwyl-Irvine et al., 2021). For each trial, all cubes were equidistant from the center of the display, thus reducing the likelihood that participants would simply select the closest cube to their current position. However, after a participant had made their first interaction, the distance of other cubes from their current position was no longer equidistant. As such, a participant may have been influenced to interact with cubes that were closer to their current position and adopt a “nearest next” strategy. Therefore, an additional factor was included in the model which measured whether the next closest cube to the previous interaction was either a heavy or light cube. Model effects and their corresponding CIs and Bayes factors can be found in Table 3 and descriptive statistics in Fig. 4.

**Table 3:** Model effects and Bayes factors—Likelihood of selecting a light cube second

*Parameter*	*Estimate*	*CIs*	*R-Hat*	*BF*
Intercept	1.36 (0.29)	**0.79, 1.95**	1.00	**585.01**
Presence (Absent–Present)	0.30 (0.24)	−0.16, 0.78	1.00	0.52
Trial Index	0.63 (0.13)	**0.37, 0.89**	1.00	**1.92×10**^**3**^
Next Closest Object Type (Heavy–Light)	1.03 (0.22)	**0.60, 1.47**	1.00	**3.20×10**^**3**^
Presence × Trial Index	0.11 (0.26)	−0.40, 0.61	1.00	0.28
Presence × Next Closest Object Type	−0.07 (0.41)	−0.86, 0.74	1.00	0.41
Trial Index × Next Closest Object Type	−0.15 (0.24)	−0.63, 0.33	1.00	0.29
Presence × Trial Index × Next Closest Object Type	0.39 (0.44)	−0.48, 1.25	1.00	0.66

*Note*. CIs = credible intervals; BF = Bayes factor; bolded CI values = CIs that did not pass through zero; bolded BF values = BF >3.20. Values in parentheses represent the associated standard error values. Effects were deemed reliable if CIs did not pass through zero and BF >3.20

Within this analysis, two main effects were observed. First, participants became more likely to select a light cube as their second interaction as the experiment progressed. Next, we found a strong effect of next closest object type on the likelihood of examining a light cube second. Here, participants were more likely to examine a light cube second if the next closest cube to their current position was also a light cube. However, it is worth noting that even on trials where the next closest cube was not a light cube, participants were still ~60–70% more likely to examine a light cube over a heavy cube. In line with our predictions, these findings further highlight the impact of effort on interaction order.

#### Number of faces viewed

**Fig. 5:**
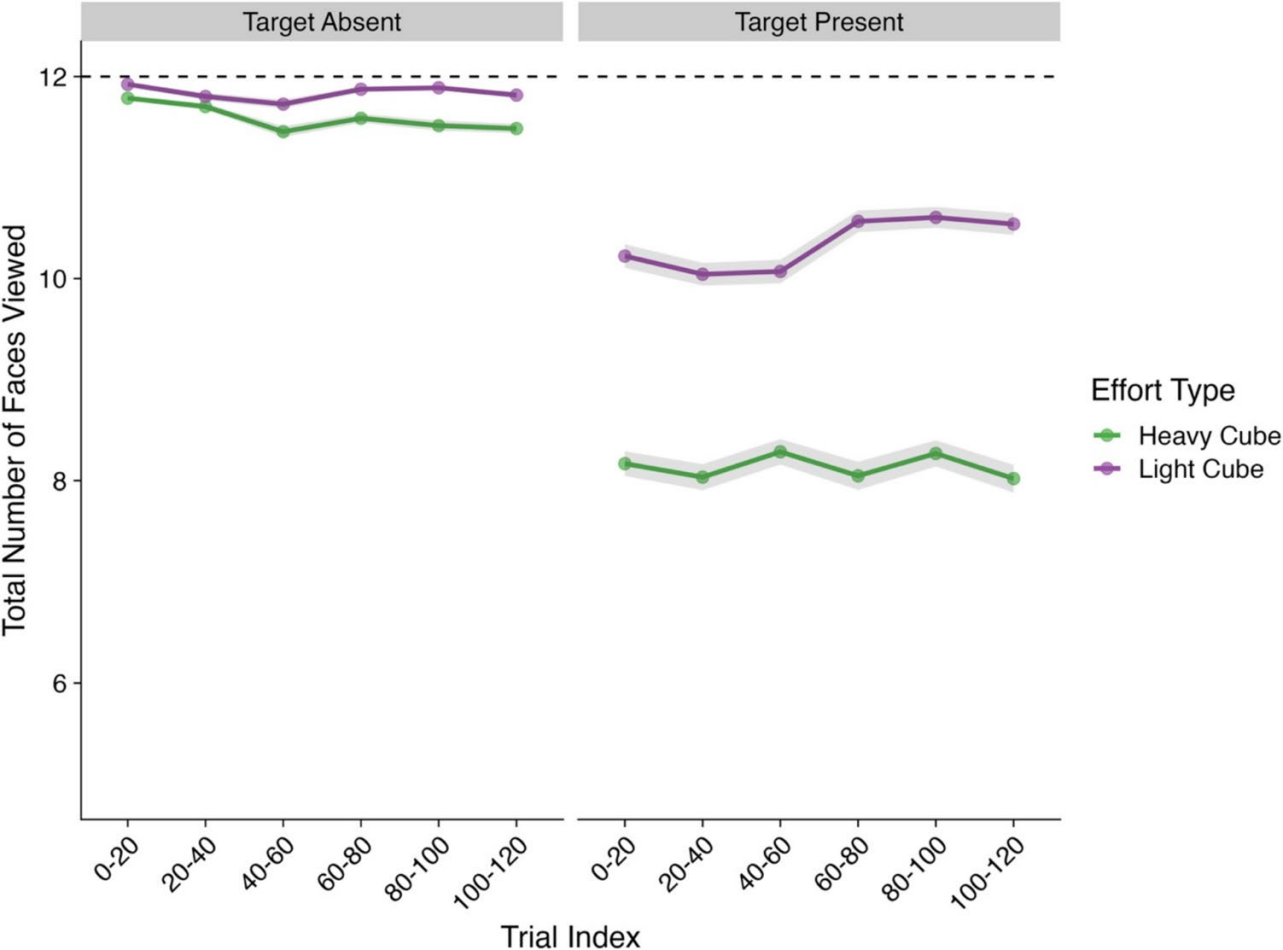
Total number of faces viewed ~ effort type (Experiment 1). *Note*. Shaded areas represent ±*SE*. Trial Index has been binned into increments of 20 trials for the purposes of visualization only. *SE* = standard error. Total viewed faces are summed and averaged for each cube type. Dashed line indicates the max number of potential faces a participant could view for each cube type. (Color figure online)

Our final analysis focused on how exhaustive participants were as they searched the displays. We measured this in terms of the number of cube faces participants viewed across each trial. For an exhaustive search, we would expect a participant to have viewed six faces per cube, or 12 faces per cube type (i.e., heavy vs. light). Model effects and their corresponding CIs and Bayes factors can be found in Table 4 and descriptive statistics in Fig. 5.

**Table 4:** Model effects and Bayes factors—Total number of faces viewed per cube type

*Parameter*	*Estimate*	*CIs*	*R-Hat*	*BF*
Intercept	2.34 (0.01)	**2.32, 2.35**	1.00	**Inf**
Presence (Absent–Present)	−0.27 (0.01)	**−0.29, −0.24**	1.00	**7.26×10**^**20**^
Trial Index	0.00 (0.01)	−0.01, 0.01	1.00	0.01
Effort Type (Heavy–Light)	0.10 (0.01)	**0.08, 0.13**	1.00	**3.32×10**^**10**^
Presence × Trial Index	0.03 (0.01)	**0.00, 0.05**	1.00	0.17
Presence × Effort Type	0.19 (0.02)	**0.15, 0.24**	1.00	**9.18×10**^**06**^
Trial Index × Effort Type	0.03 (0.01)	**0.01, 0.05**	1.00	0.36
Presence × Trial Index × Effort Type	0.03 (0.02)	−0.02, 0.07	1.00	0.05

*Note*. CIs = credible intervals; BF = Bayes factor; bolded CI values = CIs that did not pass through zero; bolded BF values = BF >3.20. Values in parentheses represent the associated standard error values. Effects were deemed reliable if CIs did not pass through zero and BF >3.20

For this analysis, we observed main effects for presence and effort type, both of which were subsumed by a Presence × Effort Type interaction. Post hoc contrasts were conducted to further understand this interaction. Overall, we observed a clear difference between the number of faces viewed between heavy and light cubes. In target-absent trials, this effect was extremely small, with a Bayes factor below the 3.20 cutoff (*Estimate* = −0.02, *lower CI* = −0.03, *upper CI* = −0.01, *BF*_*10*_ = 2.14), however, in target-present trials, this difference was much more substantial (*Estimate* = −0.24, *lower CI* = −0.26, *upper CI* = −0.22, *BF*_*10*_ = 1.77×10^32^). Put simply, participants viewed on average ~2 less cube faces for heavy cubes compared with light cubes but only within target-present trials. Since participants predominantly examined the light cubes first, this reduction in search exhaustiveness was likely a result of participants finding the target before needing to reveal the additional faces of the heavy cubes.

### Discussion

In Experiment 1, participants engaged in an interactive search for a target *T* shape attached to the side of virtual cubes. Cubes were interacted with by clicking and dragging on them with a computer mouse, which resulted in a rotation of the selected cube. On each trial, two of the four potential cubes were made to be physically effortful to interact with by reducing their sensitivity to the computer cursor. Our reasons for doing so were twofold. First, it is generally understood that there are many attributes that contribute towards attentional selection whilst searching (Awh et al., 2012; Wolfe, 2021). Second, previous literature investigating the role of physical effort and energy expenditure has been shown to influence a range of different behaviors (Anderson et al., 2025; Klein-Flügge et al., 2016; Kurniawan et al., 2010; Morel et al., 2017; Prévost et al., 2010; Steelman et al., 2011; Wang et al., 2021; Wickens, 2014, 2015). As such, we believed that when individuals are given a way to associate specific colors with increased physical effort, their attentional selection will become biased by this information with the overarching goal of reducing energetic expenditure.

We found strong evidence in favor of the notion that the effort associated with examining a given object indeed biased attentional selection within interactive searches. Put simply, participants focused on the easy-to-examine light cubes first and then moved towards the more difficult-to-examine heavy cubes later in each trial. This focus on the easy-to-examine cubes grew as the trials progressed. These findings were in line with our predictions. Due to the increased effort required to rotate heavy cubes, we expected participants to search these cubes less exhaustively and thus view fewer of their faces throughout a trial. However, we only observed this in target-present trials. In target-absent trials, participant remained exhaustive regardless of the effort condition.

The pattern of these findings provides two main takeaways from this experiment. The first is that the effort required to interact with objects appears to be a very strong driver of attentional selection indeed. It is worth noting here that within the first 20 trials, the likelihood of selecting a light cube first was ~70%. As such, the bias towards light cubes was learned and applied almost immediately. This is further supported by the fact that when the next closest cube was not a light cube, the likelihood of selecting a light cube was still ~70–75%. Here, participants would still travel the extra distance to ensure that their second interaction was to the next available light cube. The second is that the increased effort was not enough to deter participants from still examining the heavy cubes. Here, if participants could not find the target on the light cubes, they would still exhaustively examine the heavy cubes.

## Experiment 2: Patch value

In Experiment 2, we conducted a further study to determine whether perceived patch value influenced attentional selection during interactive search. To do so, we again asked participants to rotate and search through sets of virtual cubes for a *T* shape embedded onto the side of one of the cubes. Half of the cubes were made to be “information-rich” by embedding a shape to each of their six faces, and the remaining half were made to be “information-poor” by attaching a shape to only one of their six faces (hereafter we refer to these as *rich* and *poor* cubes for brevity).

We proposed that our interactive search task be further conceptualized as a foraging task for visual information (Bella-Fernández et al., 2022; Nahari & El Hady, 2025). With this in mind, the previous foraging literature suggests that individuals should become optimal in their strategies and prioritize patches that are more likely to contain high quantities of the resources they are foraging for (Bremset Hansen et al., 2009; Cain et al., 2012; Fryxell, 1991; Van Beest et al., 2010). As such, we predicted that participants would be more likely to examine the rich cubes before they examined the poor cubes. Likewise, since uncovering the additional blank faces on poor cubes would not result in any new information being uncovered, we further predicted that, compared with rich cubes, participants would be less exhaustive when searching through poor cubes and overall would stop interacting with poor cubes following the reveal of their stimulus.

### Methods

All methodological details for Experiment 2 are identical to Experiment 1, except where described below.

#### Ethical approval

Ethical approval was given for Experiment 2 by the University of Southampton’s Ethics Committee on 26 September 2023 (ERGO NUMBER: 95398.A1).

#### Participants

As with Experiment 1, a priori power analyses were carried out using the *simr* packing in R (Green & MacLeod, 2016) on pilot data from 15 participants. Power analyses were conducted for each dependent variable being analyzed. These analyses revealed that for a power level of 0.80, a minimum sample size of ~40 participants was required.

A total of 45 participants were recruited from the University of Southampton. Of this sample, ages ranged from 18 to 21 years (*M* = 19.04 years, *SD* = 1.31), with 91.11% being women, 6.67% men, and 2.22% nonbinary.

#### Stimuli and apparatus

The cubes used for Experiment 2 differed slightly from Experiment 1. We used four different types of cubes: rich and poor cubes containing only distractor *L* shapes, and rich and poor cubes containing either five *L* shapes and a single target *T* shape for rich cubes or a single target *T* shape for poor cubes. Each cube face had either a single shape attached to them or nothing at all. The same color contingencies from Experiment 1 were also used for Experiment 2. Within each trial, two of the four cubes were always poor and the other two were rich. All stimuli were evenly split between information types.

#### Design and procedure

The procedure for Experiment 2 was identical to Experiment 1 with the only difference being the stimuli used. A typical trial is depicted in Fig. 1B.

### Results

#### Data cleaning

All data underwent the same preplanned cleaning procedures as Experiment 1 before any analyses were carried out (see Table 1).

#### Analytic approach

For the most part, the same analytic approach from Experiment 1 was used for Experiment 2. All analyses used the same coding methods and model structures as Experiment 1. It is however important to note that the likelihood of selecting a light cube was changed to be the likelihood of selecting an *information-rich* cube. Likewise, the model factor effort type was changed to information type (rich, poor), and the model factor next closest cube type was changed from light and heavy to poor and rich. In comparison with Experiment 1, we conducted one additional analysis on the time it took participants to stop rotating poor cubes following stimulus reveal. For this analysis, stop times were recorded in ms and log transformed. A Gaussian distribution with the log transformed stop times were used to model effects for this measure.

#### Response accuracy and response times

Overall, for target-absent trials, participants had high accuracy with few false alarms (*M* = 0.99, *SD* = 0.09) and completed the trials within a reasonable time (*M* = 13,298.53 ms, *SD* = 5,308.53 ms). For target-present trials, participants had good accuracy (*M* = 0.96, *SD* = 0.20) and completed the trials at a reasonable pace (*M* = 6,137.18 ms, *SD* = 4,719.52 ms). We carried out no further analyses on response accuracy or response times. The remaining analyses focused on the order of interactions and search exhaustiveness.

#### First interaction choice

**Fig. 6:**
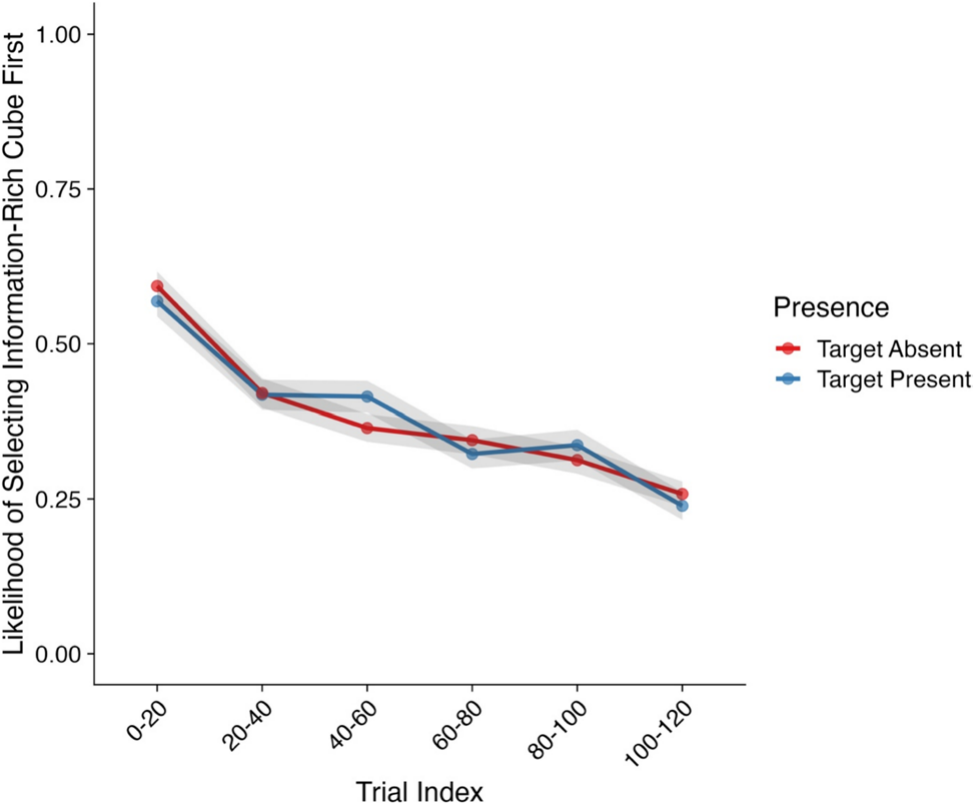
Likelihood of selecting an information-rich cube first (Experiment 2). *Note*. Shaded areas represent ±*SE.* Trial Index has been binned into increments of 20 trials for the purposes of visualization only. *SE* = standard error. (Color figure online)

Our first analysis focused on the likelihood of a participant selecting a rich cube as their first interaction of a trial. Model effects and their corresponding CIs and Bayes factors can be found in Table 5 and descriptive statistics in Fig. 6.

**Table 5:** Model effects and Bayes factors—Likelihood of selecting an information-rich cube first

*Parameter*	*Estimate*	*CIs*	*R-Hat*	*BF*
Intercept	0.36 (0.18)	0.01, 0.71	1.00	0.50
Presence (Absent–Present)	0.01 (0.13)	−0.24, 0.26	1.00	0.13
Trial Index	−.10 (0.07)	**−1.23, −0.96**	1.00	**1.08×10**^**20**^
Presence × Trial Index	−0.01 (0.14)	−0.27, 0.26	1.00	0.14

*Note*. CIs = credible intervals; BF = Bayes factor; bolded CI values = CIs that did not pass through zero; bolded BF values = BF >3.20. Values in parentheses represent the associated standard error values. Effects were deemed reliable if CIs did not pass through zero and BF >3.20

We observed an extremely strong effect of trial index on the likelihood of selecting a rich cube. Here, participants became substantially less likely to select a rich cube first as the experiment progressed. As can be seen in Fig. 6, regardless of trial type, participants started with a probability of selecting a rich cube of ~0.60 and finished with a probability of ~0.25. This finding was the polar opposite of what we predicted.

#### Second interaction choice

**Fig. 7:**
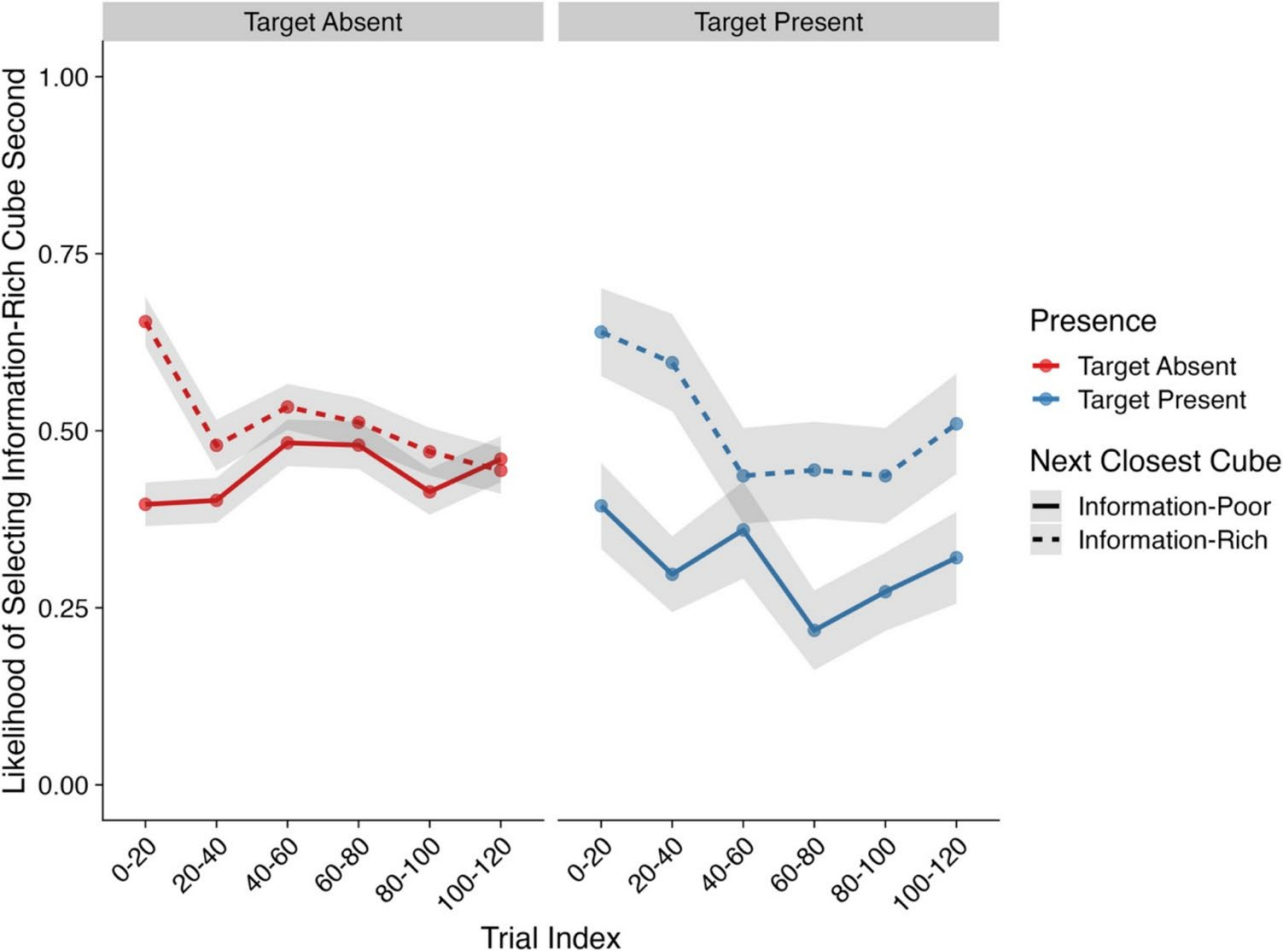
Likelihood of selecting an information-rich cube second (Experiment 2). *Note*. Shaded areas represent ±*SE*. Trial index has been binned into increments of 20 trials for the purposes of visualization only. *SE* = standard error. (Color figure online)

Our next analysis focused on the likelihood of participants selecting a rich cube as their second interaction. To recap, as with Experiment 1, following an initial interaction, cubes were no longer equidistant from a participant’s current area of attention. As such, we again included an additional model factor which measured whether the next closest cube to the previous interaction was either a poor or rich cube. This set of analyses only included data from trials where participants had not located the target during their previous interaction. Model effects and their corresponding CIs and Bayes factors can be found in Table 6 and descriptive statistics in Fig. 7.

**Table 6:** Model effects and Bayes factors—Likelihood of selecting an information-rich cube second

*Parameter*	*Estimate*	*CIs*	*R-Hat*	*BF*
Intercept	−0.01 (0.16)	−0.32, 0.31	1.00	0.05
Presence (Absent–Present)	−0.10 (0.21)	−0.52, 0.32	1.00	0.23
Trial Index	−0.33 (0.10)	**−0.53, −0.14**	1.00	**25.07**
Next Closest Object Type (Rich–Poor)	1.06 (0.18)	**0.70, 1.43**	1.00	**4.71×10**^**5**^
Presence × Trial Index	−0.32 (0.19)	−0.70, 0.06	1.00	0.76
Presence × Next Closest Object Type	0.36 (0.35)	−0.32, 1.04	1.00	0.59
Trial Index × Next Closest Object Type	−0.34 (0.18)	−0.70, 0.03	1.00	0.99
Presence × Trial Index × Next Closest Object Type	0.46 (0.35)	−0.23, 1.14	1.00	0.82

*Note*. CIs = credible intervals; BF = Bayes factor; bolded CI values = CIs that did not pass through zero; bolded BF values = BF >3.20. Values in parentheses represent the associated standard error values. Effects were deemed reliable if CIs did not pass through zero and BF >3.20

For this analysis, we observed two main effects: next closest cube type and trial index. First, participants were overall more likely to select a rich cube as their second interaction if the next closest cube to their previous interaction was also a rich cube. Next, when accounting for the effects of next closest cube type, overall, participants became less likely to select a rich cube as their second interaction as the experiment progressed. This was again not in line with our predictions.

#### Number of faces viewed

**Fig. 8:**
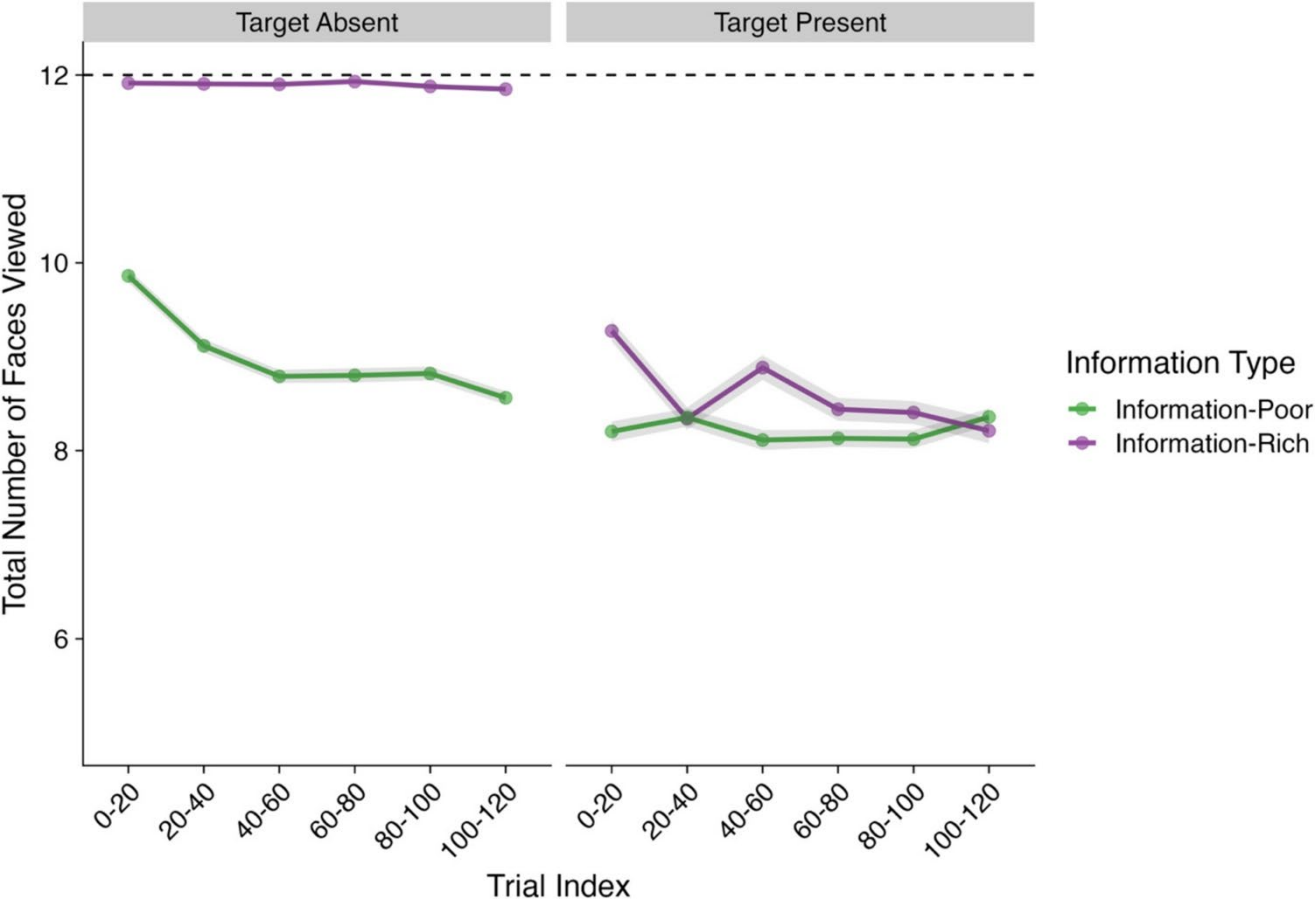
Number of faces viewed per cube type (Experiment 2). *Note*. Shaded areas represent ±*SE*. Trial index has been binned into increments of 20 trials for the purposes of visualization only. *SE* = standard error. Total viewed faces are summed and averaged for each cube type. Dashed line indicates the max number of potential faces a participant could view for each cube type. (Color figure online)

Our next analysis focused on a participant’s search exhaustiveness as measured by the number of cube faces that a participant viewed throughout a trial. Model effects and their corresponding CIs and Bayes factors can be found in Table 7 and descriptive statistics in Fig. 8.

**Table 7:** Model effects and Bayes factors–Number of faces viewed per cube type (Experiment 2)

*Parameter*	*Estimate*	*CIs*	*R-Hat*	*BF*
Intercept	2.26 (0.01)	**2.25, 2.28**	1.00	**Inf**
Presence (Absent–Present)	−0.22 (0.01)	**−0.24, −0.19**	1.00	**4.37×10**^**22**^
Trial Index	−0.04 (0.01)	−0.05, −0.03	1.00	**6.33×10**^**5**^
Information Type (Rich–Poor)	−0.16 (0.01)	**−0.18, −0.14**	1.00	**3.03×10**^**14**^
Presence × Trial Index	0.01 (0.01)	−0.01, 0.03	1.00	0.02
Presence × Information Type	0.11 (0.02)	**0.07, 0.15**	1.00	**2.28×10**^**3**^
Trial Index × Information Type	0.00 (0.01)	−0.03, 0.02	1.00	0.01
Presence × Trial Index × Information Type	0.15 (0.02)	**0.10, 0.19**	1.00	**8.19×10**^**5**^

*Note*. CIs = credible intervals; BF = Bayes factor; bolded CI values = CIs that did not pass through zero; bolded BF values = BF >3.20. Values in parentheses represent the associated standard error values. Effects were deemed reliable if CIs did not pass through zero and BF >3.20

We observed several effects within this analysis, all of which were subsumed by a three-way Presence × Trial Index × Information Type interaction. Post hoc contrasts and trend analyses were carried out to further understand this interaction.

These analyses showed that participants viewed substantially fewer faces of the poor cubes compared with the rich cubes in both target-absent trials (*Estimate* = 0.28, *lower CI* = 0.27, *upper CI* = 0.29, *BF*_*10*_ = 1.07×10^66^) and target-present trials (*Estimate* = 0.04, *lower CI* = 0.03, *upper CI* = 0.06, *BF*_*10*_ = 84.51). This effect, however, was dependent on how far through the experiment a participant was. For target-absent trials, as the experiment progressed, participants decreased the number of faces viewed for poor cubes (*Estimate* = −0.08, *lower CI* = −0.10, *upper CI* = −0.06, *BF*_*10*_ = 6.94×10^6^). In contrast, in target-present trials, as the experiment progressed, participants gradually viewed fewer cube faces for the rich cubes until there was no longer a difference between poor and rich cubes (*Estimate* = −0.07, *lower CI* = −0.09, *upper CI* = −0.04, *BF10* = 633.69).

Put simply, participants viewed ~2 fewer faces from the poor cubes compared with the rich cubes. However, this difference became more pronounced as the experiment progressed for target-absent trials and much less pronounced for target-present trials. This was in line with our predictions.

#### Time to stop interacting following reveal

**Fig. 9:**
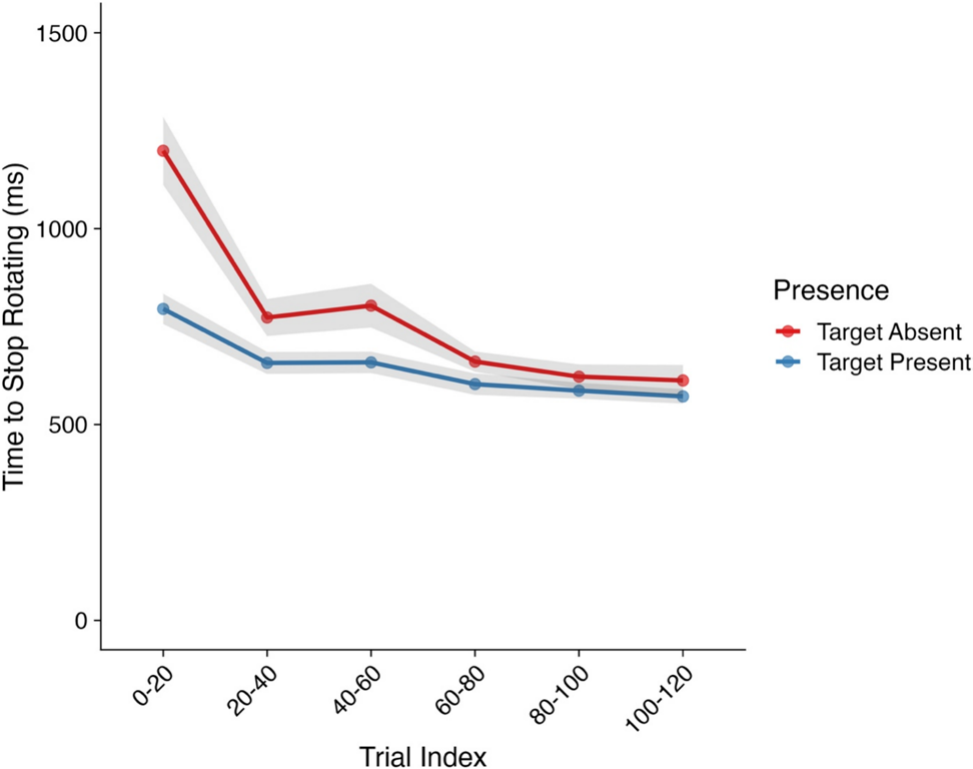
Time to stop interacting following reveal. *Note*. Shaded areas represent ±*SE*. Trial index has been binned into increments of 20 trials for the purposes of visualization only. *SE* = standard error. (Color figure online)

Finally, we examined the time it took participants to stop rotating poor cubes following stimulus reveal. Our goal here was to determine whether participants would become optimal in their searching by not continuing to interact with a poor cube following the reveal of the stimulus. As such, this analysis focused only on interactions with poor cubes where the stimulus was not visible at the start of the trial. Model effects and their corresponding CIs and Bayes factors can be found in Table 8 and descriptive statistics in Fig. 9.

**Table 8:** Model effects and Bayes factors—Time to stop interacting following reveal

*Parameter*	*Estimate*	*CIs*	*R-Hat*	*BF*
Intercept	6.31 (0.04)	**6.22, 6.39**	1.00	**8.46×10**^**178**^
Presence (Absent–Present)	−0.04 (0.03)	−0.11, 0.02	1.00	0.09
Trial Index	−0.11 (0.01)	**−0.13, −0.09**	1.00	**2.09×10**^**14**^
Presence × Trial Index	0.07 (0.02)	**0.04, 0.11**	1.00	**43.80**

*Note*. CIs = credible intervals; BF = Bayes factor; bolded CI values = CIs that did not pass through zero; bolded BF values = BF >3.20. Values in parentheses represent the associated standard error values. Effects were deemed reliable if CIs did not pass through zero and BF >3.20

Here, we observed a Trial Index × Presence interaction, which we further followed up with post hoc trend analyses. These analyses revealed that the time taken to stop interacting following stimulus reveal reduced across both target-present (*Estimate* = −0.07, *lower CI* = −0.10, *upper CI* = −0.05, *BF*_*10*_ = 5.81×10^3^) and target-absent (*Estimate* = −0.15, *lower CI* = −0.17, *upper CI* = −0.13, *BF*_*10*_ = 1.04×10^12^) trials, however, this reduction was more substantial for target-absent trials. Here, participants spent longer interacting following stimulus reveal in target-absent trials compared with target-present trials at the start of the experiment, before reducing to a similar level as target-present trials.

Overall, then, it seems that participants initially were more thorough in their searching of poor cubes in target-absent trials at the start of the experiment but learned that further examining cubes where no additional information could be gained was inefficient and costly and thus was a nonoptimal search strategy.

### Discussion

The goal of Experiment 2 was to investigate whether varying patch values could influence attentional selection within interactive search. As with Experiment 1, we had participants conduct an interactive search for a target *T* shape attached to the side of a virtual cube. Within each trial, 50% of the cubes were rich, containing six embedded shapes per cube, and the remaining 50% were poor, containing only a single embedded shape. We found that, contradictory to our predictions, participants developed an attentional bias towards examining the poor cubes before examining the rich cubes which became stronger over the course of the experiment. However, we also found that in target-absent trials participants became less exhaustive in their searching of poor cubes—as evidenced by a reduction in faces viewed and a decrease in time taken to stop interacting following stimulus reveal—but remained exhaustive for rich cubes. This suggested that, in line with our predictions, participants learned that no additional information could be gained from revealing the remaining empty faces of the poor cubes and instead learned that their energy should be spent gathering resources from the rich cubes instead.

It was surprising to find that some of our results for Experiment 2 were misaligned with our predictions. In fact, some findings were the polar opposite of what was predicted. Fortunately, there is a simple explanation that can unify and explain the results of our two experiments in a parsimonious manner, of which we shall now turn to.

## General discussion

Interactive search is commonplace within the real world yet research into the behaviors involved within interactive search has barely scratched the surface. Across these two experiments, we have identified two new forms of attentional selection that can arise during interactive search. Our predictions for both experiments were drawn from prior research into attentional selection, the influence of physical effort on behaviors, and foraging behaviors (Anderson et al., 2025; Awh et al., 2012; Wickens, 2014; Wolfe, 2021).

For Experiment 1, we predicted that participants would examine all light cubes first and then the heavy cubes after with the aim of reducing energetic expenditure, or more simply put, physical effort. Overall, we found physical effort to be an extremely strong attribute for influencing attentional selection within interactive search. Participants consistently chose to examine the light cubes before the heavy cubes, even when doing so required travelling a greater distance. However, this increased effort was not strong enough to deter participants from still exhaustively searching through heavy cubes on target-absent trials.

For Experiment 2, we predicted that participants would examine the rich cubes first followed by the poor cubes with the aim of maximizing the quantity of visual information they could obtain within any single search. However, we found the opposite: Participants developed a bias towards examining the poor cubes before the rich cubes, which grew stronger over the course of the experiment. Additionally, in target-absent trials, participants became less exhaustive in their searching of poor cubes but remained exhaustive for rich cubes.

This prompts an important question: Why were our predictions upheld for Experiment 1 but not for Experiment 2? We believe that a simpler explanation than first proposed can provide a clearer description of what might be occurring with respect to attentional selection during interactive searches. It is our belief that the unifying factor here across both experiments is, in fact, effort, and that what we have observed in our results is a consequence of a strategy aimed at minimizing both physical and cognitive effort, and subsequently, energetic expenditure.

Let us turn to Experiment 1 to begin to explain this in detail, and we can do this by considering two simple strategies. In a *difficult-first* strategy, searchers focus on the heavy cubes followed by the light cubes; in an *easy-first* strategy, searchers focus on the light cubes first followed by the heavy cubes. On half of the trials, a target is found before all cubes are examined: in fact, on average, with four cubes per trial, a target will be found by the time that two cubes have been examined in a trial. Of course, once a target has been found, the trial ends, and no more cubes are examined. Should a searcher engage in a difficult-first strategy, they will expend the highest possible amount of energy and effort per cube before finding the target; but should a searcher engage in an easy-first strategy, they will expend very little energy and effort in comparison before finding the target. It therefore makes sense that searchers focused on an easy-first strategy in Experiment 1: Doing so enabled them to conserve their effort and energy to a substantial degree.

The same argument can be used to re-cast and explain the results of Experiment 2, which were contrary to our predictions. If one does not know where a target may appear, then focusing on areas with the largest number of potential targets would be a more efficient strategy than focusing on areas with only a small number of potential targets. Thus, participants in our task were engaging in a nonoptimal strategy. However, if effort is taken into consideration, then the costs of examining the rich and poor cubes becomes important. With many more objects to examine, the rich cubes ultimately would have required more energetic expenditure and processing time than the poor cubes to fully examine. Thus, when considering the effort involved in search, a difficult-first strategy for Experiment 2 would involve examining all rich cubes before poor cubes, and an easy-first strategy for Experiment 2 would instead involve examining all poor cubes before rich cubes. Clearly, then, the search system is prioritizing effort and energetic expenditure when selecting candidate objects for detailed inspection during interactive search and overruling other considerations such as the quantity of resources a patch contains or the perceived probability that a given cube would contain a target object.

Overall, then, our two experiments combined highlight that during interactive search, searchers adopt an “easy-first” strategy, focusing on objects that can be rapidly, easily, or with little effort rejected as distractors or accepted as containing a target. In fact, though counterintuitive in some regards, our findings neatly dovetail with those reported in studies of visual search. Across a number of visual search studies, participants have been shown to use suboptimal search strategies in an attempt to reduce perceived cognitive effort (Irons & Leber, 2016, 2018; Zhang & Leber, 2024). As noted above, to our knowledge this is the first set of experiments that have been conducted with the aim of better understanding attentional selection during interactive search, and we have generated a novel set of findings regarding prioritization during interactive searches. At a theoretical level, these findings can help to better understand how, when and why regions are examined during interactive search.

It is, however, important to note that our findings may be driven not by a focus on “easy” objects first but rather by a focus on those objects that can be examined quickly.

Under this view, the longer a participant spends examining an object, the more energy and resources they must expend doing so. By prioritizing objects that can be accepted or rejected quickly (e.g., light and poor cubes), participants may therefore have been focused on reducing energetic expenditure by being more efficient with their time.^1^ Here, we did not plan on controlling for this possibility, and so drawing conclusions in this regard is beyond the scope of the current set of experiments. However, we plan on pursuing this in future research wherein the time taken to examine objects is held constant.

At a practical level, our findings also bear importance on interactive search tasks. Whether in the digital or physical world, we expect searchers to de-prioritize interactively examining objects that indicate in some way that they will require extensive effort to search. Doing so could cause these objects to not be examined at all should a searcher be under time pressure and thus terminating their searches rapidly. In our simple interactive search tasks here, performance was high and the cubes that needed more effort did not show evidence of targets being missed. However, it may be the case that in interactive search tasks that require substantially more effort overall, such as when searching for targets hidden throughout a house (Riggs et al., 2018), individuals may become more likely to avoid these objects altogether. This is indeed a possibility that we plan to examine within future experiments.

## Footnotes

1 We thank two anonymous reviewers for raising this possibility.
